# Neutrophil infiltration to the brain is platelet‐dependent, and is reversed by blockade of platelet GPIb*α*

**DOI:** 10.1111/imm.12892

**Published:** 2018-02-08

**Authors:** James A. Giles, Andrew D. Greenhalgh, Adam Denes, Bernhard Nieswandt, Graham Coutts, Barry W. McColl, Stuart M. Allan

**Affiliations:** ^1^ Faculty of Biology, Medicine and Health University of Manchester Manchester UK; ^2^ Centre for Research in Neuroscience Montreal General Hospital McGill University Health Centre Montreal QC Canada; ^3^ “Momentum” Laboratory of Neuroimmunology Institute of Experimental Medicine Budapest Hungary; ^4^ Department of Vascular Medicine University Hospital and Rudolf Virchow Centre for Experimental Biomedicine University of Würzburg Würzburg Germany; ^5^ The Roslin Institute & R(D)SVS University of Edinburgh Easter Bush, Midlothian UK; ^6^ Edinburgh Medical School UK Dementia Research Institute at The University of Edinburgh Edinburgh UK

**Keywords:** brain, inflammation, neuroinflammation

## Abstract

Neutrophils are key components of the innate immune response, providing host defence against infection and being recruited to non‐microbial injury sites. Platelets act as a trigger for neutrophil extravasation to inflammatory sites but mechanisms and tissue‐specific aspects of these interactions are currently unclear. Here, we use bacterial endotoxin in mice to trigger an innate inflammatory response in different tissues and measure neutrophil invasion with or without platelet reduction. We show that platelets are essential for neutrophil infiltration to the brain, peritoneum and skin. Neutrophil numbers do not rise above basal levels in the peritoneum and skin and are decreased (~60%) in the brain when platelet numbers are reduced. In contrast neutrophil infiltration in the lung is unaffected by platelet reduction, up‐regulation of CXCL‐1 (2·4‐fold) and CCL5 (1·4‐fold) acting as a compensatory mechanism in platelet‐reduced mice during lung inflammation. In brain inflammation targeting platelet receptor GPIb*α* results in a significant decrease (44%) in platelet‐mediated neutrophil invasion, while maintaining platelet numbers in the circulation. These results suggest that therapeutic blockade of platelet GPIb*α* could limit the harmful effects of excessive inflammation while minimizing haemorrhagic complications of platelet reduction in the brain. The data also demonstrate the ability to target damaging brain inflammation in stroke and related disorders without compromising lung immunity and hence risk of pneumonia, a major complication post stroke. In summary, our data reveal an important role for platelets in neutrophil infiltration to various tissues, including the brain, and so implicate platelets as a key, targetable component of cerebrovascular inflammatory disease or injury.

**Abbreviations**
CBA: cytometric bead array
CNS: central nervous system
IL‐1: interleukin‐1
LPS: lipopolysaccharide
TNF: tumour necrosis factor
VCAM: vascular cellular adhesion molecule‐1

## Introduction

The innate immune response provides rapid defence against infection, injury or disease. Neutrophils are the primary cellular response unit during the initial stages of these challenges and are essential for the destruction or removal of inciting stimuli.^1^ However, prolonged or excessive neutrophil‐mediated inflammation is injurious to adjacent healthy tissue in many situations, and is especially harmful during central nervous system (CNS) inflammation where capacity for repair is limited.^2^ An interaction with platelets is essential to trigger the tethering and rolling of neutrophils on inflamed venules, before their extravasation.^3^ Activated platelets attach to neutrophils via the release and surface expression of platelet P‐selectin from *α*‐granules, which binds to P‐selectin glycoprotein ligand‐1 expressed on neutrophils.^4^ After CNS injury, a dense neutrophil invasion occurs,^5^, ^6^, ^7^ and selectively abrogating neutrophil infiltration is beneficial in animal models of stroke and experimental autoimmune encephalomyelitis.^6^, ^8^ We have shown that mechanisms of neutrophil invasion following an innate immune challenge can be unique to their target tissue, allowing for tissue‐specific anti‐inflammatory interventions.^9^ This may be especially important when targeting components of the immune system that are particularly susceptible to infections, such as after stroke and spinal cord injury.^10^, ^11^ Here we assessed the contribution of platelets to neutrophil‐mediated inflammation across a variety of tissue beds, to investigate tissue‐specific mechanisms of innate immunity, as we have previously shown in the context of interleukin‐1 (IL‐1).^9^ Furthermore, we investigated whether platelet‐dependent neutrophil infiltration could be blocked, without reducing platelet numbers and increasing the risk of haemorrhage after cerebral inflammation.

## Materials and methods

### Animals

Experiments were performed on male 8‐ to 10 week‐old wild‐type C57BL/6 mice (Harlan Laboratories, Bicester, UK) under appropriate UK Home Office licences and adhered to the UK Animals (Scientific Procedures) Act 1986.

### Inflammatory challenge

#### Peritoneal inflammation model

Mice were injected intraperitoneally with 1 mg/kg lipopolysaccharide (LPS) from *Escherichia coli* O127:B8 (Sigma‐Aldrich, Dorset, UK) or vehicle (PBS) in a volume of 8 ml/kg. At 6 hr, peritoneal lavage was performed using 5 ml of lavage buffer (PBS containing 0·1% bovine serum albumin and 1 mm EDTA). Neutrophils in lavage fluid were quantified using Coulter Counter and haemocytometry measurements, combined with flow cytometry (see below).

#### Bronchoalveolar inflammation model

Mice were exposed to aerosolized LPS (2 mg/ml) or vehicle (saline) for 20 min via a nebulizer chamber. At 6 hr bronchoalveolar lavage was performed, via direct cannulation of the trachea, with 1 ml of lavage buffer.

#### Air‐pouch inflammation model

Dorsal air‐pouches were created in conscious mice as described previously.^12^ At day 7, 1 ml of LPS (1 mg/ml) or vehicle (PBS) was injected into the air‐pouch. After 6 hr air‐pouch lavage was performed using 4 ml lavage buffer.

#### Cerebral inflammation model

Animals were anaesthetized with isoflurane (3%) in O_2_ (200 ml/min) and N_2_O (400 ml/min) and placed securely in a small animal stereotaxic frame (Stoetling, Wood Dale, IL). After craniotomy, mice were injected intracerebrally with 1 μl LPS (4 mg/ml), via a glass micro‐needle (co‐ordinates from bregma: anterior–posterior −0·0 mm, lateral −2·0 mm, ventral −2·5 mm; rate = 0·5 μl/min). The micro‐needle was left *in situ* for 2 min following the injection. Mice were transcardially perfused with saline at 6 hr and brain tissue was collected for cytometric bead array (CBA) analysis or perfuse‐fixed (saline followed by paraformaldehyde 4%) at 24 hr for tissue sectioning.

#### Platelet reduction

**Figure 1:**
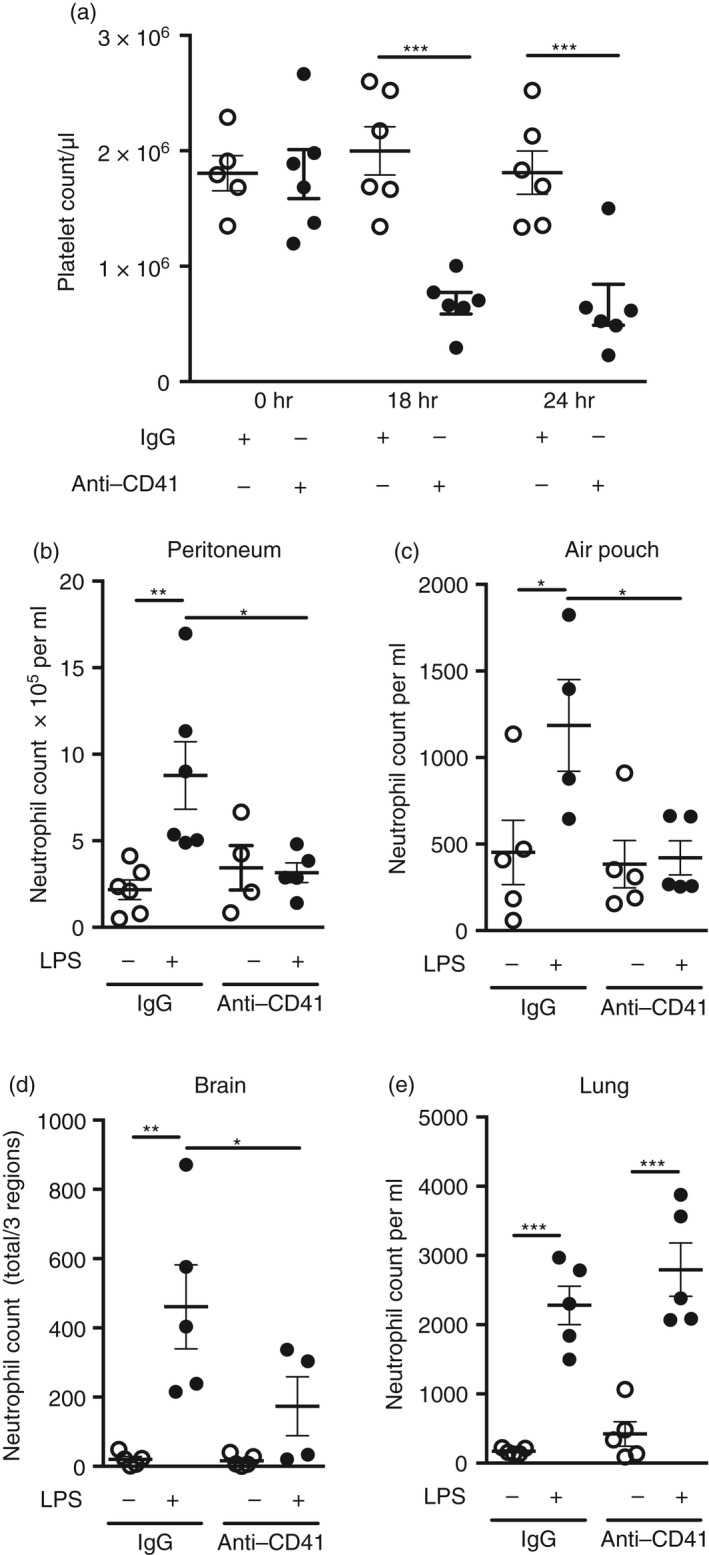
(a) Characterization of platelet reduction via anti‐CD41 antibody. Anti‐CD41 antibody or IgG control was administered intraperitoneally. Blood was sampled via tail vein sampling at 0, 18 and 24 hr post‐injection. Quantification of platelets was carried out using flow cytometry using BD TruCOUNT™ tubes. Individual data points are presented as a scatter graph with the mean ± SEM shown. ***P < 0·001; one‐way analysis of variance with Bonferroni's correction. (b–e) Neutrophil infiltration to the peritoneum, skin and brain is platelet dependent after lipopolysaccharide (LPS) ‐induced inflammation. The innate immune response was triggered by LPS challenge in four different tissues 4 hr after mice had received either anti‐CD41 antibody or control IgG (1 mg/kg), and neutrophil accumulation was measured. Neutrophil infiltration is dependent on platelets during inflammation in (b) the peritoneum, (c) subcutaneous air pouch and (d) brain. Conversely, (e) neutrophil infiltration to the lung is not affected by platelet reduction after LPS injection. *P < 0·05, **P < 0·01, ***P < 0·001; one‐way analysis of variance with Bonferroni's correction. Individual data points are presented as a scatter graph with the mean ± SEM shown.

Mice were injected intraperitoneally with anti‐CD41 antibody (1 mg/kg) or IgG isotype control (1 mg/kg) 24 hr before inflammatory challenge. Tail vein blood samples taken at 0, 18 and 24 hr post injection were analysed using flow cytometry to quantify circulating platelets. To determine the effect of the antibody on circulating leucocyte populations, cardiac blood sampled before killing at 48 hr post injection underwent flow cytometric analysis (see below) to quantify the populations of various leucocytes. Platelet numbers were reduced by ~70% (Fig. 1a) with no significant effect on circulating leucocytes (data not shown). To block platelet–endothelium interactions without any reduction in platelets, an anti‐GPIb*α* Fab fragment (p0p/B) or isotype control IgG were injected intraperitoneally (4 mg/kg) 4 hr before intrastriatal injection of LPS. Anti‐GPIb*α* treatment had no effect on numbers of circulating neutrophils (data not shown).

### Flow cytometry

Lavage fluid (200 μl) or blood (50 μl blood + 50 μl buffer: 0·1% bovine serum albumin, 1 mm EDTA in PBS) samples were incubated for 20 min with 1 : 200 rat anti‐mouse CD16/CD32 to block non‐specific Fc binding. Cocktails of fluorophore‐conjugated antibodies were added for 30 min, to detect Ly‐6G, Ly‐6C, CD45, B220, CD3, MHC‐2, Gr‐1, CD11b, CD115, CD41 and CD61. Red blood cells in samples were lysed by the addition of 450 ml FACS Lysing Solution (BD Biosciences, Oxford, UK). Absolute numbers of cells were determined through the use of TruCOUNT™ tubes (BD Biosciences), or by the addition of 50 μl fluorescent counting beads (Invitrogen, Paisley, UK). Flow cytometry was performed on a CyAn™ ADP Flow Cytometer (Dako UK Ltd, Ely, UK) equipped with 405‐nm, 488‐nm and 633‐nm lasers using summit 4.0 software (Dako UK Ltd, Ely, UK). Cell populations were determined on summit 4.0 software via positive labelling of relevant markers. For blood samples, a minimum of 1000 beads, 1000 neutrophils or 5000 leucocytes (whichever threshold occurred last) were acquired per sample. For lavage samples, a minimum of 20 000 cellular events were acquired per sample.

### Immunostaining

Total cell numbers in brain tissue sections were determined using microscopy following immunohistochemistry staining. Anti‐neutrophil (SJC4, rabbit anti‐mouse) primary antibody (1 : 50 000; kindly provided by Professor Daniel Anthony, University of Oxford, UK) was used to stain for neutrophils. Neutrophils were quantified in three regions (cortex, injection site and ventral striatum) using a 10 × 10 mm graticule at 20× magnification. Cerebrovascular activation was determined by the expression of vascular cellular adhesion molecule‐1 (VCAM‐1, goat anti‐mouse primary antibody, 1 : 250; R&D Systems, Abingdon, UK).

### Cytometric bead array

Cytokine concentrations in plasma and lavage samples were determined using mouse‐specific CBA flex sets (BD Pharmingen, Oxford, UK). CBA was used to quantify IL‐1*α*, IL‐1*β*, IL‐6, tumour necrosis factor‐*α* (TNF‐*α*), CXCL1 and CCL5 following the manufacturer's recommended protocol. Acquisition was undertaken using a BD FACSArray™ Bioanalyzer System (BD Biosciences), and results were determined using fcap array™ software (Soft Flow, New Brighton, MN).

### Statistics

Data are expressed as mean (± SEM). Differences between groups were analysed using one‐way analysis of variance with Bonferroni's correction for multiple comparisons post hoc. Differences were considered statistically significant at *P* < 0·05.

## Results

### Platelets are essential for neutrophil invasion to the peritoneum, skin and brain, but not lung after LPS‐induced inflammation

Until recently, the precise mechanism of platelet–neutrophil interaction *in vivo* during innate immune responses was unclear. However, Sreeramkumar and co‐workers recently described platelet–neutrophil dynamics in inflamed cremaster blood vessels, showing that platelets are key to initiating the process of neutrophil tethering, rolling and crawling upon vessels.^3^ To determine if platelets are required to drive innate immune responses in other tissues we stimulated inflammation with the bacterial endotoxin LPS in mice with a reduction in platelets. We used the accumulation of neutrophils as a measure of the intensity of the inflammatory reaction and assessed this at time‐points coinciding with peaks of neutrophil influx established previously for the respective tissues.^9^ LPS stimulated significant increases in neutrophil accumulation in lavage fluid from the peritoneum (4‐fold), air‐pouch (~3‐fold), lung (~13‐fold) and in the brain (~23‐fold) (Fig. 1). In contrast, after platelet reduction, neutrophil recruitment was almost completely blocked in peritoneum (96%; Fig. 1b) and air‐pouch (100%; Fig. 1c), and significantly reduced (66%) in the brain (Fig. 1d), showing platelet‐dependent neutrophil infiltration for the first time in these tissues.

Redundancy of innate immune response mechanisms is an evolutionary advantage to tissues exposed to a wide variety of pathogens, such as the lung. Here, in contrast to peritoneum, air‐pouch and brain, which are exposed to lower pathogenic load, we saw no effect of platelet reduction on neutrophil invasion after LPS‐induced inflammation in the lung (Fig. 1e), showing platelet‐independent mechanisms of neutrophil invasion.

Lipopolysaccharide injection in the peritoneum resulted in a significant increase (~2·5‐fold) in the number of circulating neutrophils at 6 hr after injection, with anti‐CD41 treatment having no effect on this increase (data not shown). In contrast, LPS, when administered in the air‐pouch, lung and brain, did not affect circulating neutrophil numbers at 6 hr (data not shown).

### Up‐regulation of CXCL1 and CCL5 in the inflamed lung counterbalances the effects of platelet reduction

**Figure 2:**
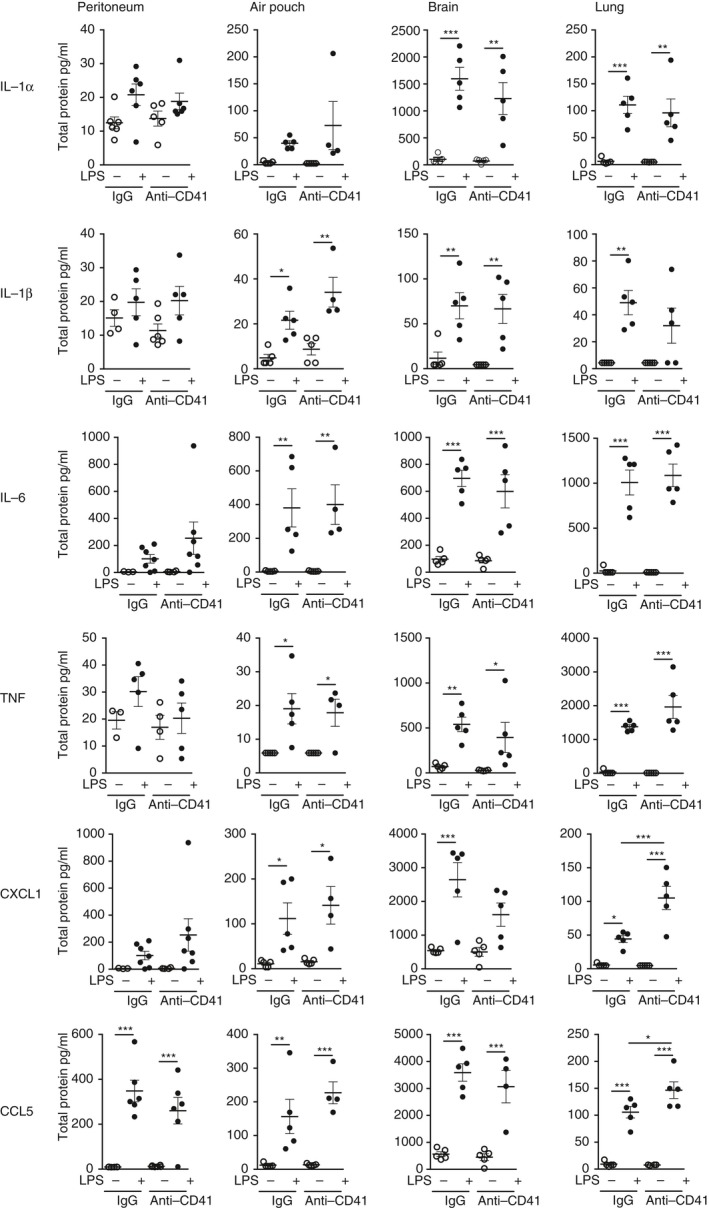
Chemokines CXCL1 and CCL5 in the inflamed lung are up‐regulated as compensatory mechanisms in response to platelet reduction. Concentrations of cytokines interleukin‐1*α* (IL‐1*α*), IL‐1*β*, IL‐6, tumour necrosis factor (TNF) and chemokines CXCL1 and CCL5 were measured in lavage fluid of peritoneum, air pouch and lung, or homogenized brain tissue after lipopolysaccharide (LPS) challenge, in mice that had received either anti‐CD41 antibody or control IgG. LPS induced a similar profile of inflammation across all tissue sites and was unaffected by platelet reduction in peritoneum, air pouch and brain. During inflammation, platelet reduction induced a significant increase in CXCL1 and CCL5 in lung lavage fluid. *P < 0·05, **P < 0·01, ***P < 0·001; one‐way analysis of variance with Bonferroni's correction. Individual data points are presented as a scatter graph with the mean ± SEM shown.

To further investigate tissue‐specific mechanisms at each site of inflammation, the cytokines IL‐1*α*, IL‐1*β*, IL‐6, TNF and chemokines CXCL1 and CCL5, which are important for neutrophil recruitment,^13^ were measured with or without platelet reduction. LPS induced a similar profile of inflammation across all tissue sites (Fig. 2). The inflammatory mediators measured were unaffected by platelet reduction in the peritoneum, air‐pouch and brain (Fig. 2). In contrast, with platelet reduction during lung inflammation, which failed to attenuate neutrophil recruitment, we found significant increases in the neutrophil chemoattractant CXCL1 (2·4‐fold), and CCL5 (1·4‐fold) in lung lavage fluid in animals with reduced platelets compared with IgG controls, and a non‐significant increase in the pro‐inflammatory cytokine TNF (Fig. 2).

### Targeting of platelet GPIbα reduces neutrophil infiltration to the brain

**Figure 3:**
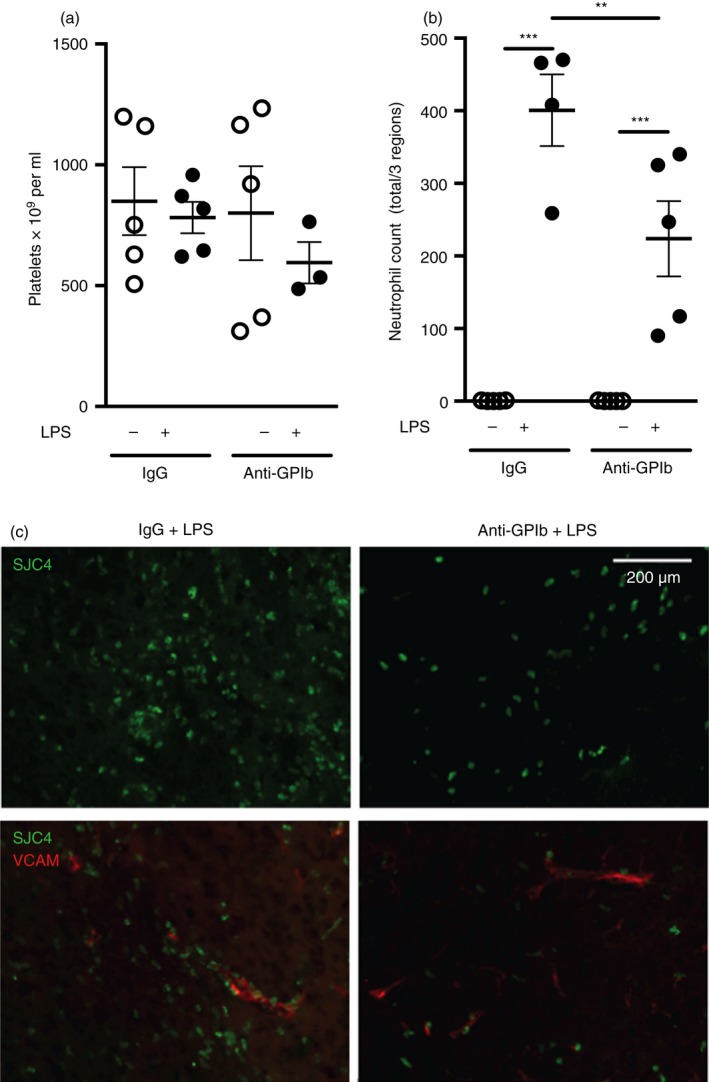
Non‐depleting targeting of platelets reduces neutrophil infiltration to the brain. Anti‐GPIb*α* antibody or IgG control was administered (4 mg/kg intraperitoneally) 4 hr before lipopolysaccharide (LPS) ‐induced brain inflammation. Anti‐GPIb*α* antibody had no effect on numbers of circulating platelets compared with IgG‐injected controls (a) yet significantly reduced the number of neutrophils in brain tissue compared with IgG control during inflammation (b). Representative immunofluorescence staining of reduced neutrophil numbers in the brain striatum following LPS injection in the presence of the anti‐GPIb*α* antibody versus IgG control, which were not accompanied by any change in cerebrovascular activation (vascular cellular adhesion molecule‐1 staining) (c). **P < 0·01, ***P < 0·001; one‐way analysis of variance with Bonferroni's correction. Individual data points are presented as a scatter graph with the mean ± SEM shown. Scale bar = 200 μm. [Colour figure can be viewed at http://wileyonlinelibrary.com]

The innate immune system is essential for host defence but excessive or prolonged neutrophil‐mediated inflammation is also associated with injury and disease, especially in the brain.^2^ The above data provide evidence for platelet‐dependent neutrophil infiltration in three different tissue beds. However, depleting platelets as an approach to reduce inflammation has important limitations due to increasing vulnerability to haemorrhage. Therefore, to assess whether platelet‐dependent neutrophil infiltration could be blocked, in a manner with less potential systemic effects and therefore more relevant therapeutically and without reducing platelet numbers, we administered anti‐GPIb*α* antibody in the context of LPS‐induced brain inflammation. Anti‐GPIb*α* antibody has been shown previously to protect mice from ischaemic brain injury in an experimental stroke model without an increase in bleeding complications.^14^ Here, anti‐GPIb*α* antibody had no effect on numbers of circulating platelets compared with IgG‐injected controls (Fig. 3a) yet significantly reduced (44%) the number of neutrophils in brain tissue after LPS (Fig. 3b,c). We saw no effect of the anti‐ GPIb*α* antibody on endothelial activation, as assessed by VCAM‐1 staining (Fig. 3c).

## Discussion

Neutrophil invasion to the brain is significantly reduced in mice lacking IL‐1,^9^ as it is here in mice with reduced numbers of platelets. Furthermore, we have shown previously that platelet‐derived IL‐1 drives endothelial activation *in vitro*, suggesting a convergence of brain‐derived and platelet‐derived IL‐1 effects on the endothelium.

These data replicate findings from Sreeramkumar *et al*.,^3^ where only LPS plus an anti MHC‐I antibody produced a strong enough lung inflammation to be attenuated by platelet intervention, as LPS‐induced inflammation alone was unaffected by blocking of platelet activity. Together, these findings provide further evidence for tissue‐specific‐mechanisms of innate immunity and highlight the flexibility of the lungs in dealing with pathogen‐driven inflammation.

These data suggest the implementation of compensatory mechanisms specific to the lung in the absence of platelets, which would be advantageous to a site of such pathogenic exposure and explain the maintenance of neutrophil recruitment, despite platelet reduction.

Targeting neutrophil invasion during CNS injury may have therapeutic benefit as we, and others, have shown neutrophil‐mediated neurotoxic effects after stroke.^6^, ^15^ Indeed, neutrophils have neurotoxic effects on neurons *in vitro*,^16^ an effect that appears dependent on phenotypic changes that occur during neutrophil cerebrovascular infiltration.^17^ Targeting various aspects of platelet activation reduces stroke injury.^3^, ^14^, ^18^ This, together with platelet‐dependent neutrophil infiltration mechanisms^3^ and the relevance of these in different tissues shown here, suggests that platelet‐targeted therapies may be beneficial after CNS injury.

A potential limitation of any immune‐modulatory approach to treating acute CNS inflammatory conditions is the potential for increased risk of systemic infectious complications, notably in conditions such as stroke and head trauma. Pneumonia is the most common cause of infection in these patients and as the innate immune response in the lung is platelet‐independent, in contrast to the brain, this may offer a relatively targeted approach to inhibiting damaging CNS inflammation without overly compromising respiratory anti‐microbial innate immunity. Platelets adhere to hypoxic endothelial cells by binding of their GPIb*α* receptor to von Willebrand factor on the endothelial surface.^19^ Targeting this interaction may limit the time spent by platelets at the endothelium and reduce the number of physical interactions with neutrophils, while concomitantly reducing the ability of platelets to activate the endothelium, which is partly responsible for driving cerebrovascular inflammation.^20^ Therefore, targeting of the platelet GPIb*α* receptor is a potential therapeutic strategy for reducing neutrophil‐mediated CNS injury.

In conclusion, we show here that platelets are essential for neutrophil extravasation to inflammatory sites, but that this is dependent on specific tissue location. We show that platelets are essential for neutrophil infiltration to the peritoneum, skin and brain, but not the lung, where compensatory mechanisms allow for greater flexibility when dealing with pathogen. We also specifically show that platelet‐mediated neutrophil invasion to the brain is dependent upon the receptor GPIb*α*, which can be targeted to limit excessive inflammation while retaining platelet numbers and reducing the risk of haemorrhage in the brain.

## Author contributions

JG, AG, BM and SA conceived the study and designed experiments. JG, AG, AD, GC and BM performed experiments and analysed data. BN provided essential tools, reagents and expertise. BM and SA co‐ordinated the study. AG, BM and SA wrote and edited the manuscript. All authors read and approved the final manuscript.

## Disclosures

The authors declare no conflict of interest.
